# Soil-transmitted Helminth infection in the Tiko Health District, South West Region of Cameroon: a post-intervention survey on prevalence and intensity of infection among primary school children

**DOI:** 10.11604/pamj.2018.30.74.15676

**Published:** 2018-05-29

**Authors:** Egbe Sarah Balle Tabi, Esum Mathias Eyong, Eric Achidi Akum, Jesper Löve, Samuel Nambile Cumber

**Affiliations:** 1 Department of Microbiology and Parasitology, Faculty of Science, University of Buea, Buea, Cameroon; 2 Cameroon Society of Epidemiology (CaSE) P.O BOX, 1411, Yaoundé, Cameroon; 3 Health Education and Research Organization (HERO) Cameroon; 4 Department of Biochemistry and Molecular Biology, Faculty of Science, University of Buea, Buea, Cameroon; 5 Section for Epidemiology and Social Medicine, Department of Public Health, Institute of Medicine (EPSO), the Sahlgrenska Academy at University of Gothenburg, P.O Box 414, SE, 405 Gothenburg, Sweden

**Keywords:** Helminths, infection, soil transmitted, school children, Cameroon

## Abstract

**Introduction:**

Soil-transmitted helminths (STH) infection remains a public health problem in sub-Saharan Africa with children being most vulnerable. STH infection may result in impairment, permanent disability or death. Annual mass deworming has been implemented in the Tiko Health District (THD), however, no study has assessed the current prevalence of STH infection. This study aimed to determine the prevalence, intensity of STH infections and associated risk factors among school children.

**Methods:**

Two months after the school deworming exercise, 400 children were sampled from 10 schools in THD. Stool samples were collected and analyzed using the Kato-katz technique. Data on socio-demographic and behavioral factors were collected using questionnaires. Data were analyzed using SPSS and intensity of infection categorized following WHO recommendations. Descriptive data were calculated with frequencies (n) and proportions (%), prevalence and 95% confidence interval calculated for gender and age respectively. Differences in prevalence for socio-demographic characteristics and behavioral variables were calculated with Chi square (χ^2^). Independent sample t-test was used to compare the means in the number of eggs in feces between male and female school children.

**Results:**

The prevalence of STH was 1% (95% CI: 0.02-1.98). *Ascaris lumbricoides* was the only STH species detected and all cases were of low intensities. The arithmetic mean egg intensity was 3.1egg per gram of faeces. Rates of infection were similar between gender and age. Site of defecation showed an association with STH infection (χ^2^ = 13.63, p=0.03).

**Conclusion:**

These findings suggested a low prevalence of STH infection which could be explained by the prior deworming of children, modification in environmental and behavioral factors. Questions on effectiveness of annual mass deworming in achieving STH elimination targets need to be investigated further.

## Introduction

Soil-transmitted helminths (STH) infection is caused by different species of parasitic nematode worms. These parasites which include the roundworm (*Ascaris lumbricoides*), the whipworm (*Trichuris trichiuria*) and 2 hookworms (*Necator americanus* and *Ancylostoma duodenale*) are spread principally through contact with feces of infected people and penetration of hookworm larvae which thrive in warm and moist soil of most tropical and subtropical countries [1]. These helminthes are collectively referred to as Geohelminths and usually co-infect their host [2]. Worldwide, about 2 billion people are infected with STHs, which is equivalent to about 24% of the world's population [3] of which 300 million suffer associated severe morbidity and even death. Amongst the STHs, *Ascaris lumbricoides, Trichuris trichiura* and Hookworms still infects 807, 604 and 576 million people respectively. Globally, *A. lumbricoides, T. trichiura* and hookworm is estimated to cause the loss of 1.817, 1.006 and 0.97 million Daily Adjusted Life Years (DALYs) respectively. The majority of DALYs were lost in Southeast Asia (47%) and sub-Saharan Africa (23%) [4]. Approximately 7.6 million children aged 1 to 15 years are at risk of infection with STH in Cameroon [3]. It was estimated that over 30.7 million African School-Aged Children (SAC) were infected with *Ascaris lumbricoides*, 36.5 million with *Trichuris trichiura* and 50 million with hookworms [5]. It is further estimated that more than 10 million people are infected with intestinal worms [6]. Morbidity from STHs is directly linked to worm burden within an individual and hence the greater the number of worms in the infected person, the greater the severity of disease. The burden of this disease is heavy within the low and middle income countries and its mainly attributed to poor sanitation and lack of adequate water supplies [3]. These infections contribute significantly to the vulnerability of our rural population [7]. Children harbor the highest prevalence and intensities of STH infection, and are very vulnerable to the effects of these parasitic infection [8, 9]. Infections with these parasites results in micronutrient deficiencies, malnutrition, poor cognitive function, mental retardation, poor school performance and absenteeism. Hookworms also have the ability to cause chronic intestinal blood loss resulting in anaemia.

Symptoms associated with STH infections are generally unspecific and subtle, hence they often go unnoticed or considered a normal condition by affected individuals, or are treated as symptoms of other diseases that might be more common in a given setting. Hence, it is conceivable that the true burden of STH infections is underestimated by assessment tools relying on self-declared signs and symptoms as is usually the case in population-based surveys [10]. WHO control interventions are based on the periodic administration of antihelminthic drugs to groups of people at risk, supported by the need for improvement in sanitation and health education. WHO recommends annual treatment in areas where the prevalence rate of soil-transmitted helminthiases is between 20% and 50% and a bi-annual treatment in areas with prevalence rates of over 50%. The global target is to eliminate morbidity due to soil-transmitted helminthiases in children by 2020 which is achieved by School Based Deworming (SBDW). Parasitological indicators used to assess impact of the treatment program include the prevalence of the infection, egg reduction rate (ERR), cure rate and the parasitic load [11] which is the main epidemiological index used to determine the level of soil-transmitted helminthes infection [12]. In public health, reduction in the number of eggs passed in faeces after drug administration is used to monitor the efficacy of the drug used against parasitic disease [13]. Cameroon adopted a strategic plan for the control of Soil Transmitted Helminthes in 2004 and from 2007, routine annual SBDW with Mebendazole has been implemented nationwide to both enrolled and non-enrolled school aged children. This is aimed at reducing the community prevalence of the disease, the intensity of infection within an individual and its associated complications [6]. The Tiko Health District (THD) has implemented several rounds of School Based Deworming with Mebendazole for STH infections. However, no study has been done to determine the prevalence, intensity and risk factors of STH infections in the THD although a known important factor in STH treatment is reinfection.

## Methods

**Figure 1:**
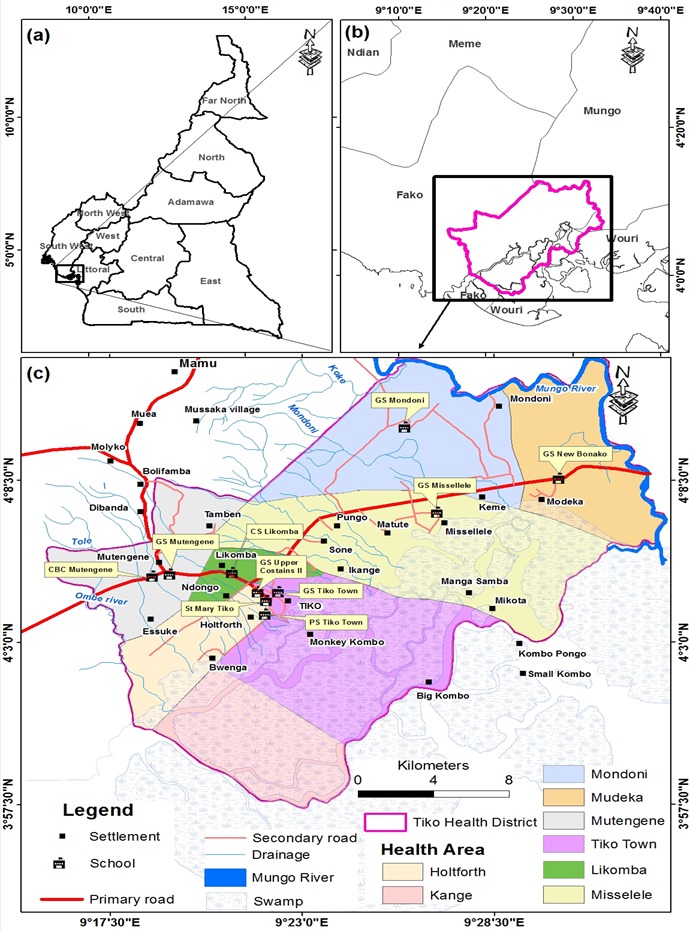
Geographic location of primary schools selected for the study in the Tiko Health District

Study area & population: This study was carried out in the THD, located in Fako Division of the South West Region of Cameroon. The district has a total surface area of 484 km^2^ and located between Longitude 8.6°10'E and Latitude 4°5.2'N. Tiko has a coastal equatorial climate with daily temperatures ranging from 28°C to 33°C. Soil types include the sandy alluvial and volcanic with high agricultural potentials. The main water courses in the Tiko municipality include River Mungo, the Ombe River, Ndongo and Benyo streams which empty into the Atlantic Ocean. The main activity of the population is trading, fishing, livestock and industrial agriculture. The THD is bounded in the North by Buea, South by Bonaberi, West by Limbe and East by Dibombari. The THD is headed by the District Medical Officer (DMO) and it is made up of 8 Health Areas (HA) namely; Holforth, Kange, Likomba, Mutengene, Mondoni, Mudeka, Missellele and Tiko Town (Figure 1). The THD has 84 primary schools including public, private lucrative and private confessional schools (IBE Tiko statistics-2016). It has a total population of 147,423 and 12.02% (17,722) of this population are primary school children. This study comprised of primary school children aged 5-15years in the THD who were present at the time of the study.

Study design, inclusion criteria: A cross sectional study was carried out in May 2016, two months after the annual school based deworming exercise to determine the current prevalence and intensity of STH infections and its associated risks factors among primary school children in Tiko Health District. Children were included in the study if their parents/legal guardians gave their consent by signing the informed consent forms and if the children gave their assent and were voluntarily willing to participate in the study. Children who had taken any anti parasitic drug less than 1 month prior to the study, who were sick or suffering from severe medical conditions and who did not have the ability to produce stool samples were excluded from the study.

Sampling method and sample size determination: In order to reduce cost and improve sampling efficiency, a stratified cluster sampling technique was used to recruit the study participants. The 84 primary schools found in the THD are unevenly distributed among the 8 (HAs). In HAs with less than 7 schools, just one primary school was randomly selected while in HAs with more than 7 schools, two primary schools were randomly selected. Random selection was done by writing the name of each school with respect to the HA on a separate piece of paper, which was then placed in a box and thoroughly mixed before selection. A simple random sampling technique was applied by blindly picking one or two papers where needed and the name of the selected school(s) written in a field note book. A total of 10 schools were selected and informed parental consents were given to all children present from grade 1 to 6, for information and written approval for their children to participate in the study. The sample size for the study was calculated using a prevalence 43.82% of STH reported by Kimbi and others (2012) in a rural population (Ekona) in the South West region of Cameroon [14]. This was done using the formula; n=Z2pq/d2 Where; n=the expected sample size, p= prevalence of STH infection in school children from previous studies, q= 1-p, d=margin of error, z= 1.96 (95% CI). Estimated sample size for the study was 378.29, adding a non- compliance rate of 5% gave us 397.2. Thus a minimum sample size of 397 was needed for the study.

**Data collection**: Prior to the start of the study, visits were made to all the randomly selected schools. Letters of Administrative Authorizations were presented to the various Head Teachers, and the purpose/benefits of the study explained to both the teachers and children. Children present on the day before sample collection were given consent forms to take to their parents/legal guardians to read and consent by signing. Children were identified by individual codes and their names written separately in a notebook for the purpose of the return of their results. This study included the collection of both qualitative and quantitative data.

**Questionnaire**: Each child was questioned separately on his/her socio-demographic and behavioral factors. This was done to avoid influence from friends. Qualitatively, a structured questionnaire was used to collect information on demographic characteristics and risk factors such as age, sex and parental occupation, hand washing practices, walking barefoot, presence of toilets and its usage and types of water sources available for domestic purposes.

**Stool sample collection and examination**: Children whose parents/guardians had granted a written informed consent were instructed on how to collect stool samples. They were provided with an A4 sheet, toilet roll and a clean well labelled container for stool collection. The children were instructed to defecate on the A4 sheet to avoid contamination from the toilet environment and then using the spoon inside the stool container, pick up a small portion of the faeces and transfer to the stool container. Stools were kept cool in a flask with ice packs and transported to the CMA Holforth laboratory for macroscopic and microscopic analysis. After this exercise, children washed their hands with water and detergent which was provided to them.

**Laboratory analysis**: Stool samples were immediately transported in a cool flask to CMA Holforth laboratory for analysis. Upon arrival at the Laboratory, macroscopic examination of stool samples was done. Quantitatively, stools were examined using the Kato-Katz technique. Kato-Katz thick smears were examined 30-60 minutes after preparation to avoid over clearing of Hookworm eggs, using the 10x and 40x objectives of the microscope. For quality control, a random sample of positive and negative kato-katz thick smears were re-examined on a daily basis to confirm previous results.

**Statistical analysis**: First, descriptive data were calculated with frequencies (n) and proportions (%) of socio demographic characteristics. Test for normality (Kolmogorov) was analyzed to establish if the variables were normally distributed. In order to investigate the overall research question, prevalence and 95% confidence interval were calculated for gender and age respectively. Thereafter, differences in prevalence for socio-demographic characteristics and behavioral variables were calculated with Chi square (χ^2^), p<0.05. Independent sample t-test was used to compare the means in the number of eggs in faeces between male and female school children. All data analysis were performed in SPSS version 20.

**Ethical considerations**: Ethical approval was obtained from the Institutional Review Board of the Faculty of Health Science, University of Buea, Cameroon. Administrative authorizations were gotten from the Regional Delegation of Public Health, Regional Delegation of Basic Education in the South West Region of Cameroon. Additional administrative approvals were gotten from the District Medical Officer of the Tiko Health District, Inspectorate of Basic Education Tiko and Head teachers of the selected schools. A brief talk was given to the school children on the objectives, protocol and benefits of the study. Participation in the study was voluntary, children's assent was sought and only the children whose parents consented by signing the informed consent form were recruited as study participants. Participants had equal chances of participating in the study and had the right to withdraw at any time during the study without being questioned. Confidentiality was ensured by giving serial numbers to each participant who were used in both the questionnaire and the stool container. Participant's names were only used for the purpose of issuing of individual results. Stool samples were collected by trained Medical Laboratory Scientists with experience in phlebotomy. After stool collection, it was ensured that the children washed their hands with a hand washing detergent to avoid contamination. At the end of the study, all the selected schools were re-visited and the results of each participant was given. Head teachers of schools with STH infected children were properly informed and had the responsibility of informing the parents of these children, to consult at the Health Centre to ensure proper management of their clinical condition.

## Results

**Socio-demographic characteristics of the study participants**: Of the 400 study participants, 183 (45.7%) were males. Majority of the study participants, 50.7% were aged between 5-9 years and 49.3% >9 years. The median age of the study population was 9 (IQR=3), with an age range between 5-15 years spread across grade 1 to grade 6. Grades 1 had the lowest proportions (2.3%) of study participants while Grade 5 had the highest proportion of participants (40.5%). Majority of the parents of the children (36.5%) were employed by the Cameroon Development Corporation (CDC) while some were involved in business (19.5%). The least proportions were found among the parents involved in fishing (2%). A greater proportion of the participants (66.3%) lived in block houses and a least proportion of children lived in asbestos (7%) (Table 1).

**Table 1:** Socio-demographic characteristics of the study population

Socio-demographic characteristics		Frequency (n=400)	Proportions (%)
**Gender**	Male	183	45.7
	Female	217	54.3
**Age (years)**	5-9	203	50.7
	>9	197	49.3
**Grade**	1	9	2.3
	2	17	4.3
	3	88	22
	4	99	24.8
	5	162	40.5
	6	25	6.3
**Parental occupation**	Farmer	57	14.3
	Fishing	8	2.0
	Business	78	19.5
	Civil Servant	25	6.3
	CDC	146	36.5
	Unemployed	14	3.5
	Others	72	18.0
**House Type**	Cement	265	66.3
	Planck	107	26.8
	Asbestos	28	7.0
**Floor Type**	Cemented	326	81.5
	Un-cemented	74	18.5

**Prevalence of STH Infection**: Positive cases were reported from GS Upper Costains, GS New Bonako, and CS Likomba. The overall prevalence of STH infection in the 10 surveyed schools was found to be 1% (95% CI: 0.02-1.98). The specific STH found was *Ascaris lumbricoides* with a zero prevalence of the other STH species. Of the 4 children infected with Ascaris, rates of infection were similar between gender and age groups (Table 2).

**Table 2:** STH Infection status among the total study population presented per age and gender with frequencies and proportions

Socio-demographic characteristics		STH Infection Status	
		Positive, n (%)	Negative, n (%)
**Gender**	Male	2 (1.1)	181 (98.9)
	Female	2 (0.9)	215 (99.1)
	**Total**	**4**	**396**
**Age (years)**	5 to 9	2 (1)	201 (99)
	>9	2 (1)	195 (99)
	**Total**	**4**	**396**

**Figure 2:**
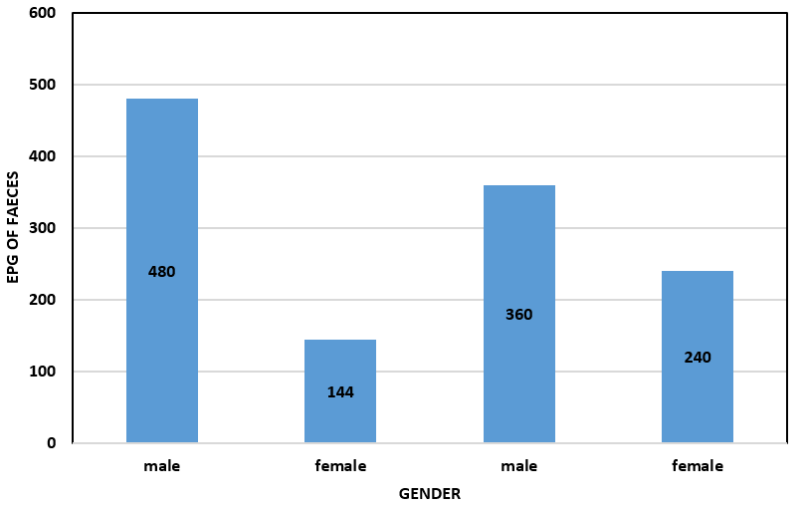
Intensity of STH infection among positive cases presented as Epg of faeces

**Intensity of STH infection**: The intensity of *Ascaris lumbricoides* infection among the school children who were found positive was characterized based on WHO classification of STH infection intensities (Table 3). The arithmetic mean egg intensity for *Ascaris lumbricoides* was 3.1 epg. Although all the four infected children (1%) had light intensity infection of *Ascaris lumbricoides*, males had a higher egg counts (480 epg and 360 epg) than females (240 epg and 144 epg). However, testing for difference in mean intensity between male and females, there was no statistically significant difference (t = 2.967, P= 0.097) (Figure 2).

**Table 3:** Classification of the Intensity of Soil-Transmitted Helminth infection as eggs per gram of stool

STH Infection	Severity of infection [eggs per gram of stool]		
	Mild	Moderate	Severe
Roundworms	1-4,999	5,000-49,999	≥50,000
Pinworms	1-999	1,000-9,999	≥10,000
Hookworms	1-1,999	2,000-3,999	≥4000

[Source: Pan American Organization, 2011]

**Socio-demographic risk factors associated with development of STH infection**: Examining the association of STH infection with possible socio-demographic risk factors, none of this factors were significantly associated with STH infections (P>0.05) (Table 4).

**Table 4:** Socio-demographic factors associated with soil-transmitted Helminth infection

Socio-demographic variables		Soil-Transmitted Helminths		χ2 (P-Value)
		Positive n (%)	Negative n (%)	
Gender	Male	2 (50)	181 (45.7)	0.029 (0.864)
	Female	2 (50)	215 (54.3)	
Age (years)	5-9	2 (50)	201 (50.8)	0.001 (0.976)
	>9	2 (50)	195 (49.2)	
Grade	1	0	9 (2.3)	2.535 (0.771)
	2	0	17 (4.3)	
	3	0	88 (22.2)	
	4	2 (50)	97 (24.5)	
	5	2 (50)	160 (40.4)	
	6	0	25 (6.3)	
Parents occupation	Farmer	0	57 (14.4)	9.202 (0.163)
	Fishing	0	8 (2.0)	
	Business	1 (25)	77 (19.4)	
	Civil Servant	1 (25)	24 (6.1)	
	CDC	1 (25)	145 (36.6)	
	Unemployed	1 (25)	13 (3.3)	
	Others	0	74 (18.2)	
Housing Type	Cement	3 (75)	264 (66.2)	0.334 (0.846)
	Planck	1 (25)	106 (26.8)	
	Asbestos	0	28 (7.1)	
Floor Type	Cemented	3 (75)	323 (81.6)	0.113 (0.737)
	Uncemented	1 (25)	73 (18.4)	
Toilet Present	Yes	3 (75)	364 (91.9)	1.498 (0.221)
	No	1 (25)	32 (8.1)	
Source of drinking water	Tap	4 (100)	328 (82.8)	0.828 (0.660)
	Well	0	16 (4.0)	
	Stream	0	52 (13.1)	

**Behavioral risk factors associated with development of STH infection**: Examining the association of STH infection with possible behavioral risk factors, defecation site was significantly associated with STH infection (χ^2^ = 13.633, P= 0.03) (Table 5).

**Table 5:** Behavioral factors associated with soil-transmitted Helminth infection

Behavioral variables		Soil-Transmitted Helminths		χ2 (P-Value)
		Positive n (%)	Negative n (%)	
Hand washing before eating	Always	1 (25)	165 (41.7)	0.453 (0.501)
	Sometimes	3 (75)	231 (58.3)	
Hand wash after toilet	Always	0 (0)	161 (40.7)	2.722 (0.099)
	Sometimes	4 (100)	235 (59.3)	
Defecation site	Water closet	0 (0)	66 (16.7)	13.633 (0.03)**
	Pit latrine	2 (50)	267 (67.4)	
	Bush	1 (25)	57 (14.4)	
	Streams	1 (25)	6 (1.5)	
Walk Barefoot	Always	0 (0)	3 (0.8)	0.368 (0.832)
	Sometimes	3 (75)	239 (60.4)	
	Never	1 (25)	154 (38.9)	
Eat soil	Yes	0 (0)	43 (10.9)	0.487 (0.485)
	No	4 (100)	353 (89.1)	
Bite fingers	Yes	2 (50)	241 (61)	0.202 (0.653)
	No	2 (50)	154 (39)	

## Discussion

Our study revealed an overall prevalence of 1% for STHs which is lower than that reported in other parts of Cameroon [7, 14-18]. However, a low prevalence rate (2.5%) was reported in selected rural, semi-urban and urban communities in the Mount Cameroon area [19]. A prevalence rate of 0.5% was also reported in primary school children in the Same District in Northern Tanzania [20]. Intensity of infection was generally low, consistent with the findings of Mugono and others (2014) in North-Western Tanzania who reported that in regions where STH is targeted for elimination with annual mass deworming, high worm burdens are not very common and most of the individuals infected with STH normally excrete a low number of eggs [21]. Male individuals had higher egg counts as compared to female individuals which is consistent with findings of other studies [7]. This could be attributed to variation in exposure to risk factors. The risk factors assessed in this study were selected based on the fact that transmission of intestinal parasites is related to poor sources of drinking water, hygienic practices, fecal disposal systems, socioeconomic status and existence of wide variations of parasites within human communities. In this study, the only risk factor which statistically showed an association with STH infection was defecation site where water closet shows lowest prevalence. However, this results should be interpreted with caution based on the risk of Type II errors. The prevalence and intensity of STH infection within populations has previously been linked to different factors most importantly socioeconomic, environmental, parasitic and host factors [18].

The low prevalence of STH infection in the THD may be attributed to the recent SBDW which took place 2 months before this study was carried out. Antihelminthic chemotherapy has also been integrated in the Maternal and Child Health services in Cameroon, where children under the age of five years are given antihelminthic drugs during the Mother and child health action week (MCHAW). This could also account for the observed low prevalence of STH infections in the THD because treating this age group is of importance in reducing transmission intensities. In addition, there could be environmental and behavioral factors that could have led to the sustained low prevalence of STH infections in primary school children in the THD. The positive cases of STH found in this study were of the age group 9 and 11 years old which is consistent with the findings of Mbuh and others (2012) who found out that the prevalence of STH was highest among children aged between 6 and 12 years [18]. In relation to gender, the prevalence of STH was similar in males and females which is consistent with reports of a previous study in the South West Region of Cameroon [19] but in contrast to the study carried out by Ntonifor and others (2015) in a recently established focus behind the Mount Cameroon Area who found out that the prevalence of STH was higher in males than females [7]. This study contributes to what is already known with regards to prevalence and intensity of STH infections. This study had a representation of the Health Areas in the Tiko Health District and made use of a large sample size. Limitations encountered include the inconsistent pattern of associations, use of single stool sample to identify helminth which might have led to underestimation of the prevalence of STH infection, absence of a baseline prevalence of STH making it difficult to directly link the low prevalence gotten from the study with the SBDW implemented. Factors associated with the sustained low prevalence of STH infections in the district following years of mass chemotherapy need to be thoroughly investigated, so that they can be positively reinforced. Based on the findings of this study, we would like to recommend further studies to be carried out before and after the SBDW exercise prior to any practical recommendations.

## Conclusion

Based on the findings of this study, we conclude that the prevalence of Soil-transmitted Helminth infection in school children in the Tiko Health District was 1%, all infections were of low intensity and the risk factor associated with STH infection was site of defecation.

### What is known about this topic

- The epidemiology of Soil Transmitted Helminth infection in Kekem, West-Cameroon which indicated a high prevalence of Soil Transmitted Helminth, proving that the infection still continues to pose a public health challenge to inhabitants of rural areas;
- The influence of urbanization on the prevalence and intensity of soil transmitted helminths infections in the mount Cameroon Region, which highlighted the fact that the prevalence of infection decreases with increase in the level of urbanization;
- The prevalence of geohelminths and the impact of albendazole on parasitic indices in Kotto Barombi and Marumba which suggested that geohelminths infection remains a serious health problem in school children in the Kotto Barombi focus. However, post treatment control showed a decrease in prevalence and mean parasitic load.

### What this study adds

- The effective deworming exercise being carried out in the Tiko Health District is resulting to a comparatively low prevalence and intensity of soil transmitted helminth infection;
- The importance of environmental hygiene and sanitation, most especially the use of toilets for defecation is a very important indicator in the control of soil transmitted helminth infection.
